# Ruptured Rudimentary Horn Pregnancy at 25 Weeks with Previous Vaginal Delivery: A Case Report

**DOI:** 10.1155/2012/985076

**Published:** 2012-06-06

**Authors:** Deepa V. Kanagal, Lokeshchandra C. Hanumanalu

**Affiliations:** ^1^Department of Obstetrics & Gynecology, K. S. Hegde Medical Academy, Karnataka, Mangalore 575018, India; ^2^Department of Obstetrics & Gynecology, Mysore Medical College & Research Institute, Karnataka, Mysore 570001, India

## Abstract

Unicornuate uterus with rudimentary horn occurs due to failure of complete development of one of the Mullerian ducts and incomplete fusion with the contralateral side. Pregnancy in a noncommunicating rudimentary horn is extremely rare and usually terminates in rupture during first or second trimester of pregnancy. Diagnosis of rudimentary horn pregnancy and its rupture in a woman with prior vaginal delivery is difficult. It can be missed in routine ultrasound scan and in majority of cases it is detected after rupture. It requires a high index of suspicion. We report a case of G2PlL1 with rupture rudimentary horn pregnancy at 25 weeks of gestation which was misdiagnosed as intrauterine pregnancy with fetal demise by ultrasound, and termination was attempted and the case was later referred to our hospital after the patient developed hemoperitoneum and shock with a diagnosis of rupture uterus. Laparotomy revealed rupture of right rudimentary horn pregnancy with massive hemoperitoneum. Timely laparotomy, excision of the horn, and blood transfusion saved the patient.

## 1. Introduction

Mullerian anomalies were first classified in 1979 by Buttram and Gibbons and further revised by the American Society of Reproductive Medicine in 1988. Unicornuate uterus is a type 2 classification with unilateral hypoplasia or agenesis that can be further subclassified into communicating, noncommunicating, no cavity, and no horn [1]. The incidence of uterine congenital anomalies because of Mullerian defects in the normal fertile population is 3.2%. A unicornuate uterus accounts for 2.4%–13% of all Mullerian anomalies. [2] 72–85% of the rudimentary horns are noncommunicating with the cavity [3]. Unicornuate uterus with rudimentary horn may be associated with gynecological and obstetric complications like infertility, endometriosis, hematometra, urinary tract anomalies, abortions, and preterm deliveries. Rupture during pregnancy is the most dreaded complication which can be life threatening to the mother. We report a case of ruptured rudimentary horn pregnancy in shock at 25 weeks of gestation misdiagnosed as intrauterine pregnancy and attempted termination for fetal demise.

## 2. Case Report

A 25-year-old G2P1L1 with 25 weeks of pregnancy was referred to the emergency ward of our hospital at midnight from a peripheral rural health centre with a diagnosis of rupture uterus. Our hospital is a referral hospital attached to a government medical college which mainly caters to rural population. The lady had a previous uneventful vaginal delivery of a 2.5 kg baby 3 years back. This was her second pregnancy. She had antenatal checkups at a rural primary health centre. She went for an ultrasound examination at 25 weeks of gestation due to pain abdomen and absent fetal movements. The ultrasound examination done at the peripheral centre showed an intrauterine fetal demise of 24 weeks. In view of the fetal demise, the lady was induced with misoprostol for expulsion of the fetus. By twelve hours after induction she developed hypotension, tachycardia, and hypovolemic shock. In view of these features, a diagnosis of rupture uterus was made and the patient was referred to our hospital.

On examination, the lady was in hypovolemic shock with severe pallor and rapid feeble pulse. Her blood pressure was not recordable. The abdomen was tense and distended and the uterine size was not made out. Pelvic examination revealed fullness in the fornices with cervical movement tenderness. There was no vaginal bleeding. As the patient was in shock, she was taken for immediate laparotomy after resuscitation. Her hemoglobin was 3 gm% at the time of laparotomy.

**Figure 1:**
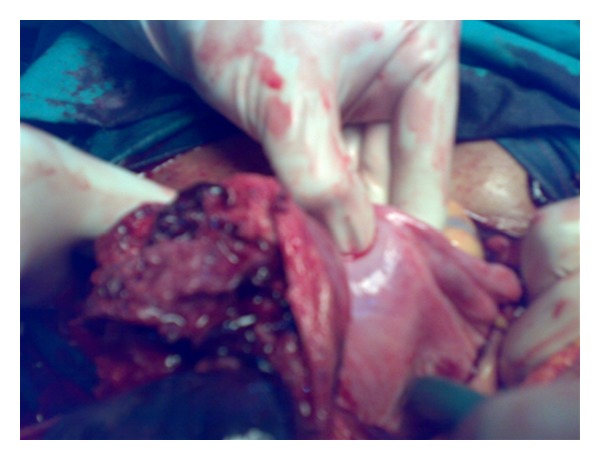
Rupture right rudimentary horn.

**Figure 2:**
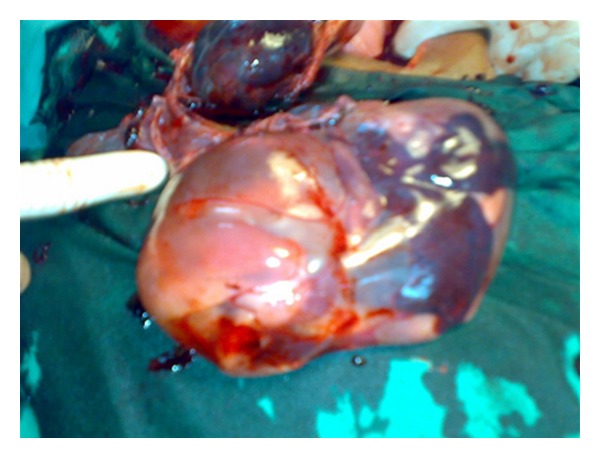
Fetus with intact sac.

**Figure 3:**
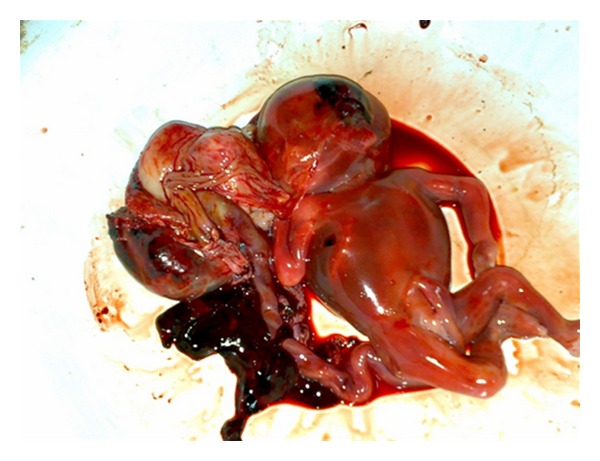
Fetus with placenta.

At laparotomy, there was a rupture of right rudimentary noncommunicating horn of a unicornuate uterus (Figure 1) with the fetus and intact sac lying free in the peritoneal cavity with a hemoperitoneum of about three litres (Figure 2). The fetus weighed about 600 grams (Figure 3). The rudimentary horn was excised. After achieving hemostasis, abdomen was closed in layers after keeping a drain. The lady was transfused with 5 units of blood. Her postoperative recovery was good. She was later investigated for urinary tract anomalies which were found to be absent. She was discharged from the hospital on the eighth postoperative day.

## 3. Discussion

 A rudimentary horn with a unicornuate uterus results due to failure of the complete development of one of the Mullerian ducts and incomplete fusion with the contralateral side.

The incidence is estimated at 1 per 100,000 to 140,000 pregnancies [3]. Pregnancy in a noncommunicating rudimentary horn occurs through the transperitoneal migration of the spermatozoon or the transperitoneal migration of the fertilized ovum [4]. The first case of uterine rupture associated with rudimentary horn was reported in 1669 by Mauriceau [5]. The timing of rupture varies from 5 to 35 weeks depending on the horn musculature and its ability to hypertrophy and dilate. 70–90% rupture before 20 weeks and can be catastrophic [6]. As the uterine wall is thicker and more vascular, bleeding is more severe in rudimentary horn pregnancy rupture [7]. Kadan and Romano described rudimentary horn rupture as the most significant threat to pregnancy and a life-threatening situation [8]. Maternal mortality rate before 1900 was reported to be 47.6%. Rupture of the horn is still common but no case of maternal death has been published since 1960 [9]. Early diagnosis of the condition is essential and can be challenging. Ultrasound, hysterosalpingogram, hysteroscopy, laparoscopy, and MRI are diagnostic tools [10]. Fedele et al. have found ultrasonography to be useful in the diagnosis [11]. But the sensitivity of ultrasound is only 26% and sensitivity decreases as the pregnancy advances [12]. It can be missed in inexperienced hands as in our case. Tubal pregnancy, cornual pregnancy, intrauterine pregnancy, and abdominal pregnancy are common sonographic misdiagnosis [13]. There are no definitive clinical criteria to detect this life-threatening condition in case of emergency, and diagnosis can be difficult because the enlarging horn with a thinned myometrium can obscure the adjacent anatomic structures.

Tsafrir et al. reported 2 cases of rudimentary horn pregnancy found in the first trimester by sonography and confirmed by MRI. They outlined a set of criteria for diagnosing pregnancy in the rudimentary horn [14]. They are (1) a pseudo pattern of asymmetrical bicornuate uterus; (2) absent visual continuity tissue surrounding the gestation sac and the uterine cervix; (3) presence of myometrial tissue surrounding the gestational sac. Nonetheless, most of the cases remain undiagnosed until it ruptures and present as emergency. Cases of late and false diagnosis leading to uterine rupture have been reported. Use of labor induction agents for termination of pregnancy in a rudimentary horn is unsuccessful and can lead to rupture of the horn. Samuels and Awonuga reported rupture after use of misoprostol due to misdiagnosis [15]. This happened in our case too. Nonresponders to induced abortion should be investigated with a high index of suspicion. Buntungu et al. reported a rudimentary horn pregnancy in a 6th gravida with all previous normal deliveries with a diagnosis of intrauterine fetal demise in this pregnancy where induction with misoprostol failed leading to the suspicion of ectopic pregnancy [16].

Primary strategy of management of rudimentary horn is surgical removal [9]. There are instances of early diagnosis and laparoscopic excision of rudimentary horns. Dicker et al. removed a small rudimentary horn through the suprapubic laparoscopic port [17]. Yoo et al. resected a pregnant horn of 5 × 5 cm laparoscopically [18]. Yahata et al. used endoscopic stapler to transect a fibrous band connecting the rudimentary horn to the uterus [19]. Medical management with methotrexate and its resection by laparoscopy is also reported. Edelman et al. showed a case detected at an early gestational week and treated successfully with methotrexate administration [20].

Immediate surgery is recommended by most after the diagnosis even in unruptured cases [12]. Removal of the horn prior to pregnancy in order to prevent complications is also advised. However, conservative management, until viability is achieved, has been advocated in few selected cases if emergency surgery can be performed anytime and if the patient is well informed [9]. A case of pregnancy progressing to the third trimester and resulting in live birth after cesarean section has been documented [21]. Renal anomalies are found in 36% of cases [12]; hence it is mandatory to further assess these women.

## 4. Conclusion

Despite advances in ultrasound and other diagnostic modalities, prenatal diagnosis remains elusive, with confirmatory diagnosis being laparotomy. The diagnosis can be missed in ultrasound especially in inexperienced hands. Precious time may be lost due to delay in diagnosis or misdiagnosis and the general condition of the person may worsen as in our case. Timely resuscitation, surgery, and blood transfusion are needed to save the patient. Proper diagnostic methods and early referral from the peripheral hospitals is needed to reduce the morbidity and mortality of the patients. There is a need for an increased awareness of this condition especially in developing countries where the possibility of detection before pregnancy or before the rupture is unlikely, and precious time is lost in shifting these women to the referral hospital.
